# Inferring the Deep Past from Molecular Data

**DOI:** 10.1093/gbe/evab067

**Published:** 2021-03-27

**Authors:** Tom A Williams, Dominik Schrempf, Gergely J Szöllősi, Cymon J Cox, Peter G Foster, T Martin Embley

**Affiliations:** 1 School of Biological Sciences, University of Bristol, United Kingdom; 2 Department of Biological Physics, Eötvös Loránd University, Budapest, Hungary; 3 MTA-ELTE “Lendület” Evolutionary Genomics Research Group, Budapest, Hungary; 4 Institute of Evolution, Centre for Ecological Research, Budapest, Hungary; 5 Centro de Ciências do Mar, Universidade do Algarve, Gambelas, Faro, Portugal; 6 Department of Life Sciences, Natural History Museum, London, United Kingdom; 7 Biosciences Institute, Centre for Bacterial Cell Biology, Newcastle University, Newcastle upon Tyne, United Kingdom

**Keywords:** phylogenetics, tree of life, substitution models, eukaryote origins, microbial evolution

## Abstract

There is an expectation that analyses of molecular sequences might be able to distinguish between alternative hypotheses for ancient relationships, but the phylogenetic methods used and types of data analyzed are of critical importance in any attempt to recover historical signal. Here, we discuss some common issues that can influence the topology of trees obtained when using overly simple models to analyze molecular data that often display complicated patterns of sequence heterogeneity. To illustrate our discussion, we have used three examples of inferred relationships which have changed radically as models and methods of analysis have improved. In two of these examples, the sister-group relationship between thermophilic *Thermus* and mesophilic *Deinococcus*, and the position of long-branch Microsporidia among eukaryotes, we show that recovering what is now generally considered to be the correct tree is critically dependent on the fit between model and data. In the third example, the position of eukaryotes in the tree of life, the hypothesis that is currently supported by the best available methods is fundamentally different from the classical view of relationships between major cellular domains. Since heterogeneity appears to be pervasive and varied among all molecular sequence data, and even the best available models can still struggle to deal with some problems, the issues we discuss are generally relevant to phylogenetic analyses. It remains essential to maintain a critical attitude to all trees as hypotheses of relationship that may change with more data and better methods.

**Significance**

Phylogenetics can help to test hypotheses of ancient relationships, but the model used is critically important in recovering historical signal. Here, we review three case studies that demonstrate how improvements in phylogenetic modeling can lead to radical change in the inferred trees and their biological interpretations. Model selection is a fundamental step in phylogenetic analysis, and trees are hypotheses that may change with new data and better methods.

## Introduction

Phylogenetic trees provide a framework for understanding the evolution of life’s diversity. However, the phylogenetic methods and data that are best to use when attempting to infer relationships between the major groups of life are still being keenly debated (Pisani et al. 2015; Williams et al. 2020). In this review, we discuss some of the key issues that can cause phylogenetic inferences to be misled when insufficient attention is given to the fit between the patterns in molecular sequence data and the models used to analyze them.

The first trees inferred from molecular data were inferred using maximum parsimony (MP) or distance-matrix approaches coupled with fairly simple site and time-homogeneous models of sequence evolution (Jukes and Cantor 1969; Tavaré 1986). A site- and time-homogeneous model assumes that the process of evolution remains constant over the sites of the alignment and the branches of the tree. These assumptions are unrealistic because sites evolve under different functional constraints (Liberles et al. 2012), and real molecular data are heterogeneous across the branches of a tree and across the different sites and genes of sequence alignments. For example, orthologous sequences from different species often manifest very different amino acid or nucleotide compositions because they are evolving in different ways. A failure to accommodate heterogeneity in phylogenetic models results in model misspecification (that is, the use of an inadequate substitution model) and can potentially lead to the recovery of trees that display spurious phylogenetic relationships, sometimes with strong support (Felsenstein 1978; Woese et al. 1990; Kuhner and Felsenstein 1994; Swofford et al. 2001; Philippe et al. 2011).

Over time, new phylogenetic models have been developed to try and better accommodate the kinds of heterogeneity that affect real data. These models contain parameters that describe the process of evolution in terms of the exchangeability of different nucleotides or amino acids, the long-term expected frequencies of the different character states, and the underlying phylogenetic tree. To learn about the evolutionary process and tree, the models are fit to the data using maximum likelihood (ML) or Bayesian methods. Since these analyses are performed in a probabilistic framework, standard tools from statistics can be used to choose and evaluate models and trees, and to determine which parameters most improve the fit of the model to the data. A clear strength of the model-based approach is that models can be updated as statistical practice and computational methods improve or as new aspects of the evolutionary process are discovered. Although it is not expected that any model will ever fit real data perfectly, empirical work and simulations have shown that the newer models generally perform better than MP and simpler site and time-homogeneous models at recovering the correct tree under a variety of realistic conditions (Foster 2004; Ho and Jermiin 2004; Jermiin et al. 2004; Lartillot et al. 2007; Schrempf et al. 2020).

As methods have improved, it is not surprising that some previously accepted relationships have been challenged and replaced by new trees and new hypotheses. Here, we discuss three case studies where model misspecification appears to have misled the field. For two of these examples—the relationship between *Thermus* and *Deinococcus*, and the place of Microsporidia parasites in the eukaryotic tree—there is now consensus about what the true tree should look like, so we can evaluate the performance of different methods at mitigating problems. In the third example, the topology of the tree of life, the hypothesis currently considered to be best-supported by phylogenomics (Williams et al. 2013, 2020; Eme et al. 2017) is different to the classical view (Woese and Fox 1977; Woese et al. 1990) of relationships between bacteria, archaea, and eukaryotes. An important take-home message from these case studies is that real data sets often contain different types of heterogeneity affecting different parts of an alignment or tree. Although individual types of variation can be accommodated by carefully chosen models, joint effects can be extremely difficult to diagnose or overcome, especially when a mixture of long external branches and short internal branches are present. Since all of these phenomena are pervasive in studies investigating early evolution, the issues we discuss have relevance beyond our chosen examples. It remains essential to maintain a critical attitude to trees as hypotheses that may change with more data and better methods.

### Different Sites in Genes, and the Same Genes in Different Species, Can All Evolve at Different Rates

Early models for inferring trees from nucleic acid sequences such as the Jukes and Cantor model (JC69; Jukes and Cantor 1969; see table 1 for an overview of all of the models we discuss and apply in this review) assumed that all sites evolve at the same rate. But in real sequence data, different sites evolve at different rates due to variation in site-specific selective constraints. Some sites have not changed across large evolutionary distances whereas others evolve at high rates (Fitch and Margoliash 1967; Dickerson 1971; Uzzell and Corbin 1971; Miyamoto et al. 1996). When performing phylogenetic inference, among-site rate variation (ASRV) can be beneficial because it means that individual gene and protein sequences can contain information about different levels of phylogenetic relationships. Fast-evolving sites are useful for resolving close relationships but may quickly lose signal through overwriting by new substitutions, whereas slowly evolving sites can retain signal for more distant relationships (Woese et al. 1991; Penny et al. 2001; Foster et al. 2009). Across-site rate variation (ASRV) can be modeled using a gamma distribution whose shape parameter is estimated from the data (Uzzell and Corbin 1971; Golding 1983; Yang 1993, 1996; which we denote with +G below) or by estimating a set of site rate categories directly (Yang 1995; Susko et al. 2003; Kalyaanamoorthy et al. 2017). This generally improves the fit between model and data and can help to ameliorate phylogenetic artifacts such as long-branch attraction (LBA).

**Table 1:** Features of the Phylogenetic Methods Discussed in This Article

Model	Across-Branch Compositional Heterogeneity	Across-Site Compositional Heterogeneity	Reference	Notes
JC69	No	No	Jukes and Cantor (1969)	Equal character (nucleotide) frequencies and exchange rates
JC2	No	No	Jukes and Cantor (1969)	(As above, for binary data)
TIM2	No	No		AC/AT and CG/GT exchange rates the same.
GTR (General time reversible)	No	No	Tavaré (1986)	Exchange rates and compositions inferred from the data
NDCH (node-discrete compositional heterogeneity)	Yes (node-discrete)	No	Foster (2004)	More than one branch composition vector; compositions can change at speciation events
NDCH2	Yes (node-discrete)		Foster (2004); Williams et al. (2020)	Each branch has its own composition, constrained by a hyperparameter
CAT	No	Yes	Lartillot and Philippe (2004)	Nonparametric modeling of site-specific compositions; all exchange rates equal (Poisson)
CAT+GTR	No	Yes	Lartillot and Philippe (2004)	As CAT but with different exchange rates among characters
CAT+BP	Yes	Yes	Blanquart and Lartillot (2008)	As CAT but with composition changing at discrete breakpoints (BP) that can be placed anywhere on the tree
WAG	No	No	Whelan and Goldman (2001)	Fixed exchange rates between amino acids, inferred from a database of sequence alignments
LG	No	No	Le and Gascuel (2008)	Fixed exchange rates between amino acids, inferred from a database of sequence alignments
LG+C60	No	Yes	Le and Gascuel (2008); Quang et al. (2008); Yang (1995)	Fixed exchange rates and 60-site compositions inferred from alignment database; weights of mixture components inferred during analysis.
UDM128 (Universal distribution mixture)	No	Yes	Schrempf et al. (2020)	128 fixed site compositions inferred from alignment database; weights of mixture components inferred.
GHOST	No	Yes	Crotty et al. (2020)	Models heterotachy via a mixture of substitution processes and branch lengths across sites
LogDet distance	Yes	No	Lockhart et al. (1994); Lake (1994); Steel (1994)	Additive distance measure consistent with a model in which compositions can change anywhere on the tree
COaLA	Yes (node-discrete)	No	Groussin et al. (2013)	Models branch heterogeneity using a small number of parameters that describe the main axes of compositional variation in a data set

Note.—There are several add-ons to the basic models, including +F (amino acid frequencies inferred from the data, rather than those specified by the model); +I (models a proportion of invariant sites); +G (ASRV modeled with a mixture of gamma-distributed rates across sites, usually approximated by four or eight rate categories); +R*x* (ASRV modeled with a mixture of *x* free rates that are not constrained to be drawn from a gamma distribution).

LBA occurs when two or more long branches in a tree group together irrespective of their true relationships, and was first recognized as a problem for maximum parsimony (Felsenstein 1978). Long-branched taxa have a higher probability of sharing the same character state because of parallel or convergent changes along long branches. Since outgroup sequences are often on long branches, long-branching ingroup sequences will often be attracted to the base of the ingroup (Olsen 1987; Holland et al. 2003; Shavit et al. 2007). Models that ignore ASRV will systematically underestimate the amount of change that has occurred at variable sites and may be particularly susceptible to LBA (Felsenstein 1978, 1982; Olsen 1987; Hendy and Penny 1989; Huelsenbeck 1995; Hirt et al. 1999; Tourasse and Gouy 1999; Sullivan and Swofford 2001; Swofford et al. 2001).

Conventional ASRV models assume that each site maintains its characteristic rate throughout time and in all lineages (Penny et al. 2001). In other words, some sites always evolve quickly whereas others always evolve slowly. However, early studies already demonstrated that the evolutionary rates of homologous sites in cytochrome c differed between Metazoa and Fungi (Fitch and Markowitz 1970; Fitch 1971a). The “concomitantly variable codon” or “covarion model” was proposed (Fitch and Markowitz 1970; Fitch 1971b) to explain the observed distributions of variable sites by suggesting that at any one time only a small fraction of sites are free to vary, with the identity of variable sites able to change over time and in different lineages. In the original implementations of the covarion model, sites in proteins were only allowed to shift between two states over time, either invariable (“off”) or variable (“on”), with all variable sites sharing a common substitution model and rate (Fitch and Markowitz 1970; Tuffley and Steel 1998; Penny et al. 2001). Probabilistic models implementing covarion-like processes of evolution have extended the original concept to allow sites to switch between a number of different rates as well as an invariable state as they evolve across the tree (Galtier 2001; Huelsenbeck 2002; Wang et al. 2007; Zhou et al. 2007, 2010). The property whereby the evolutionary rate of a site can vary over time and in different lineages has also been called heterotachy (Philippe and Lopez 2001; Lopez et al. 2002), and it is this term that is now generally used to describe models aiming to accommodate this apparently common property of sequence data (Kolaczkowski and Thornton 2008; Crotty et al. 2020). Simulation studies (Wang, Susko, et al. 2008) and empirical analyses (Yang 1996) suggest that failure to model ASRV or heterotachy can result in the inference of an incorrect tree. Attempts to resolve the phylogenetic position of Microsporidia provide a good example of how failing to sufficiently consider rate variation can mislead attempts to recover accurate phylogenetic relationships for extremely long-branched taxa.

### Long-Branch Attraction and the Position of Microsporidia in the Eukaryotic Tree

Microsporidia are obligate intracellular parasites of animals (Vavra and Lukes 2013) and gregarines (Mikhailov et al. 2017). They are now thought to represent highly derived fungi, a phylogenetic position supported by shared genes and cell biological traits including the presence of a chitinous cell wall (Capella-Gutiérrez et al. 2012; James et al. 2013; Bass et al. 2018). However, their molecular sequences are highly divergent compared with free-living eukaryotes (Vossbrinck and Woese 1986; Kamaishi, Hashimoto, Nakamura, Masuda, et al. 1996; Kamaishi, Hashimoto, Nakamura, Nakamura, et al. 1996), and early analyses of SSU rRNA and protein sequences using methods that did not model ASRV resolved Microsporidia near the base of the eukaryotic tree (Vossbrinck et al. 1987; Sogin et al. 1989; Leipe et al. 1993; Hashimoto and Hasegawa 1996; Kamaishi, Hashimoto, Nakamura, Masuda, et al. 1996; Kamaishi, Hashimoto, Nakamura, Nakamura, et al. 1996). These analyses provided important support for the influential Archezoa hypothesis for eukaryotic evolution, which proposed that Microsporidia and other long-branched anaerobic and parasitic protists were primitively without mitochondria, having branched from the eukaryotic tree before the mitochondrial endosymbiosis (Cavalier-Smith 1987).

The first gene trees to suggest that Microsporidia might not be early branching eukaryotes (“Microsporidia early”), were trees for alpha- and beta-tubulin sequences which suggested that Microsporidia could instead be related to fungi (“M+F”; Edlind et al. 1996; Keeling and Doolittle 1996). These data were quickly followed by discoveries that Microsporidia contained orthologs of mitochondrial (mt)Hsp70, a protein of alphaproteobacterial origin that performs essential functions inside mitochondria (Germot et al. 1997; Hirt et al. 1997; Lill 2009). In gene trees, microsporidian mtHsp70 grouped weakly with fungal orthologs, suggesting that if Microsporidia really lacked mitochondria then this was the result of secondary loss rather than primitive absence. Subsequent analyses of the largest subunit of RNA polymerase II for the microsporidians *Variomorpha necatrix* and *Nosema locustae* strongly supported M+F (Hirt et al. 1999).

The two alternative positions of Microsporidia in different gene trees provided competing hypotheses for which support could be compared as data were analyzed using better models. Hirt et al. (1999) reanalyzed the original EF-2 alignments used to place “Microsporidia early” (Hashimoto and Hasegawa 1996; Kamaishi, Hashimoto, Nakamura, Nakamura, et al. 1996) and demonstrated that this was due to a failure to model ASRV, combined with the presence of long-branch archaeal outgroup sequences. Thus, the removal of the long-branch archaeal outgroup sequences and a partial-correction for ASRV by removing the fastest evolving sites (fast site removal or FSR; Waddell and Steel 1997), gave an unrooted ingroup tree in which Microsporidia formed a clan (Wilkinson et al. 2007) with Fungi consistent with M+F (Hirt et al. 1999).

Understanding why “Microsporidia early” was recovered from EF1-alpha sequences proved more challenging, although support was reduced after FSR (Hirt et al. 1999). Subsequent work demonstrated that site rates varied between the EF1-alpha sequences of eukaryotes and Archaea (Inagaki et al. 2003; Wang et al. 2007), violating the ASRV assumption that site rates are constant over the tree and suggesting that heterotachy might be contributing to model misspecification (Stiller and Hall 1999; Inagaki et al. 2003). EF1-alpha from the microsporidian *Glugea plecoglossi* also contains many nonconservative amino acid substitutions at otherwise universally conserved positions (Kamaishi, Hashimoto, Nakamura, Masuda, et al. 1996; Hirt et al. 1999; Inagaki et al. 2004). Removal of a proportion of the sites that contributed most to the across-tree site-rate variation between eukaryotic and archaeal sequences reduced support for “Microsporidia early” but did not recover M+F (Inagaki et al. 2004).

To investigate further, Kolaczkowski and Thornton (2008) reanalyzed the EF1-alpha data set from Inagaki et al. (2004) using a mixed branch-length model for heterotachy. This mixture model incorporates site-specific changes in evolutionary rates by summing likelihoods over multiple sets of branch lengths on the same tree (Kolaczkowski and Thornton 2008). The model recovered M+F with strong support suggesting that support for “Microsporidia early” from EF1-alpha was indeed due to a failure to sufficiently model heterotachy in previous analyses. Consistent with that result, analysis of a concatenated alignment of 133 single-copy protein-coding genes using a covarion model allowing for site rate shifts across the tree (Wang et al. 2007) recovered strong support for M+F (Wang et al. 2009). By contrast, previous analyses modeling ASRV for the same data had recovered a “Microsporidia early” tree (Brinkmann et al. 2005; Wang et al. 2009).

The first studies to use models including ASRV to analyze the influential SSU and LSU rRNA data sets recovered reduced support for “Microsporidia early” but did not recover M+F (Kumar and Rzhetsky 1996; Peyretaillade et al. 1998; Stiller and Hall 1999). All of these analyses included long-branch outgroup taxa and it appears that modeling ASRV was not sufficient to eliminate LBA under these conditions. Subsequent analyses of concatenations of LSU and SSU rRNA sequences using a range of increasingly sophisticated models incorporating both ASRV and covarion-like structure also failed to consistently recover M+F in the presence of long-branch outgroups (Fischer and Palmer 2005; Cox et al. 2008; Foster et al. 2009). It thus appears to be extremely difficult to recover M+F from rRNA sequences in the presence of long-branch outgroup sequences and short internal branches, even when ASRV or covarion structure is modeled. Consistent with this conclusion, an analysis of LSU rRNA sequences from a selection of crown taxa including fungi, but excluding long-branch taxa like *Giardia* and prokaryotic outgroups, recovered M+F with weak support in an unrooted tree (Van de Peer et al. 2000). These results suggest that investigating whether the removal of long-branch outgroups affects the stability of ingroup relationships is a useful general check for difficult data sets (Van de Peer et al. 2000; Shavit et al. 2007; Cox et al. 2008).

### Compositional Heterogeneity among Sequences Can Mislead Phylogenetic Inference: The Case of *Thermus* and *Deinococcus*

Most of the substitution models in wide use, including the LG model (Le and Gascuel 2008) for amino acids and the GTR model (Tavaré 1986) for nucleotides, make the assumption that the frequencies of the 20 amino acids or four nucleotides remain constant over time in homologous sequences. But real genes and proteins do not evolve like this (Steel et al. 1993; Foster et al. 1997) and across-tree variation in nucleotide and amino acid composition is a common feature of molecular data (Lake 1994; Lockhart et al. 1994). Nevertheless, many studies continue to use models that assume stationarity of nucleotide or amino acid composition over time and this can cause sequences with similar compositions to group together regardless of their true relationships. A classic example of this phenomenon is the difficulty that homogeneous models have in recovering the correct sister-group relationship between the thermophilic and mesophilic sister taxa *Thermus* and *Deinococcus* from SSU rRNA sequences.

**Fig. 1.**
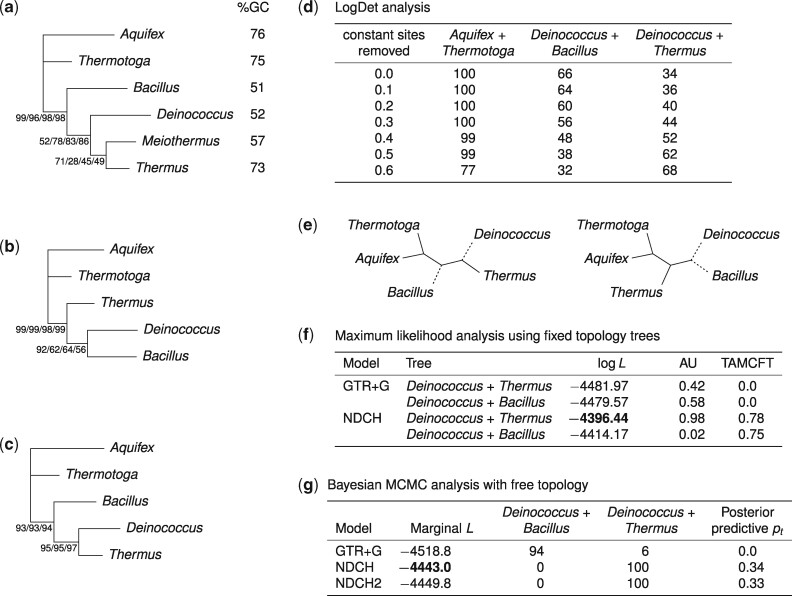
—Investigating the relationship between *Thermus* and *Deinococcus*. (*a*) Phylogeny of six taxa inferred from an alignment of SSU rRNA under maximum parsimony (MP) and three simple commonly used substitution models (JC69+G, GTR+G, TIM2+F+G; see table 1 for a description of these models and their differences). All three models assume that sequence composition is constant over the sites of the alignment and branches of the tree. A clan comprising *Thermus*, *Meiothermus*, and *Deinococcus* is recovered with moderate bootstrap support, as indicated (MP/JC69/GTR/TIM2). This is believed to be the correct tree. (*b*) When the analysis is repeated after removing the mesophile *Meiothermus ruber*, none of these methods recover the correct tree. Instead, the sequences group according to composition, with the two moderate %GC mesophiles (*Deinococcus* and *Bacillus*) forming a clan. (*c*) RY recoding of the data recovers the correct *Deinococcus*+*Thermus* tree under both MP and two ML models for two-state data (JC2+G and GTR2+G). (*d*) Distance-based analysis using the LogDet distance recovers the correct relationship (*Deinococcus*+*Thermus*) with increasing support as constant sites are progressively removed as an incremental correction for ASRV; the composition of constant sites is distinct from that of variable positions. (*e*) Arrangement of composition vectors on the (correct) *Deinococcus–Thermus* and (incorrect) *Deinococcus–Bacillus* trees in the NDCH+G model in (*f*) and (*g*). The dotted composition vector (moderate %GC) is placed on branches leading to mesophile tip taxa; the solid composition vector (high %GC) is placed on all other branches and the root. (*f*) ML analysis of the two fixed trees under the composition-homogeneous GTR+G model does not distinguish between the two trees (AU > 0.05; Shimodaira 2002), whereas the composition-heterogeneous NDCH+G model rejects the incorrect *Deinococcus–Bacillus* tree. The TAMCFT (tree and model composition fit test; Foster 2004) results indicate that the NDCH+G model, but not the GTR+G model, fits the data adequately with respect to compositional heterogeneity. (*g*) Free topology analysis using MCMC to search tree space. The NDCH+G and NDCH2+G models fit the data better (higher marginal likelihood) and provide maximal support (PP = 1) to the correct *Deinococcus*+*Thermus* tree; the GTR+G model fits worse and provides moderate support for the incorrect *Deinococcus*+*Bacillus* tree. Sequence data simulated using the parameters of the NDCH+G and NDCH2+G models are similar to the real data (posterior predictive test using the χ^2^ statistic, *P* > 0.05), but data simulated under GTR+G are not (*P* = 0), providing additional evidence that NDCH+G and NDCH2+G, but not GTR+G, adequately fit the data with respect to composition.

A relationship between *Thermus* and *Deinococcus* was originally proposed based upon similarities in their membrane lipids, cell wall composition and rRNA oligonucleotide cataloguing data (Hensel et al. 1986) and is supported by phylogenies made using broadly distributed single-copy genes (Wang and Wu 2013). Based upon the congruence between these different types of evidence, they are now classified together in a *Deinococcus*–*Thermus* phylum. However, when full-length SSU rRNA sequences were first analyzed using the JC69 model (Weisburg, Giovannoni, et al. 1989; Weisburg, Tully, et al. 1989), a sister-group relationship between *Thermus* and *Deinococcus* was only recovered when the analysis was limited to slowly evolving sequence positions where one nucleotide accounted for at least 50% of the composition. Slowly evolving positions have a lower %GC content than more variable positions and are less saturated by multiple changes. These early analyses suggested that compositional heterogeneity among sequences (that is, across the tree) might have impacted the inferred relationships among *Thermus*, *Deinococcus*, and other thermophilic and mesophilic taxa. To illustrate these effects and how they can be mitigated, we used a variety of models and data treatments (fig. 1) to analyze a small SSU rRNA data set including *Thermus* and *Deinococcus*. When considering the results from these analyses, it is worth remembering that across-tree compositional heterogeneity affects most other data sets, including those used to investigate the phylogenetic position of Microsporidia (Cox et al. 2008) and the conserved proteins used in tree of life phylogenomic studies (Williams et al. 2020).

Figure 1*a* shows the tree recovered for the SSU rRNA sequences of six taxa: *Thermus thermophilus*, *Meiothermus ruber*, *Deinococcus radiodurans*, an unrelated mesophilic bacterium *Bacillus subtilis*, and two thermophiles, *Aquifex aeolicus* and *Thermotoga maritima*, that are not closely related to *Thermus*. Their SSU rRNA %GC varies between 51 and 76 for variable positions in the alignment. *Meiothermus ruber* (Hensel et al. 1986; Embley et al. 1993; Nobre et al. 1996) previously classified as *Thermus ruber*, has a lower optimal growth temperature (∼60 °C) and its SSU rRNA sequence has a lower GC content (57%) compared with *Thermus thermophilus*. Phylogenetic analysis of the SSU rRNA data for the six taxa produces an unrooted tree whereby *Thermus* and *Meiothermus* together with *Deinococcus* are recovered as a clan (Embley et al. 1993; Wilkinson et al. 2007) with moderate bootstrap support (fig. 1). The support for what is considered to be the correct topology is obtained even though four of the six taxa fail the composition χ^2^ test individually (*P* ranging from 0.0084 to 0.044), and nine of the 15 sequence pairs also fail Stuart’s test (Ababneh et al. 2006) for marginal symmetry (*P* < 1e-6). Thus, despite not fitting the data for composition, all four methods including parsimony can recover the true tree when *Meiothermus ruber* is included. By contrast, when the analysis is repeated after removing *Meiothermus ruber* (fig. 1*b*), neither MP nor the ML models were able to recover the clanship of *Thermus* and *Deinococcus*, and the five taxa separate according to shared nucleotide composition, with the two lowest %GC taxa, *Deinococcus* and *Bacillus*, grouping together in the tree. This result clearly demonstrates that taxon sampling, that is, the inclusion of a mesophilic relative of *Thermus*, can sometimes facilitate the recovery of the correct tree even when there is an inadequate fit between the models used and the data being analyzed.

Data can also be recoded to ameliorate problems caused by across-tree compositional heterogeneity. For example, RY recoding (transversion analysis) involves recoding nucleotides as either purines (R) or pyrimidines (Y) (Woese et al. 1991; Phillips and Penny 2003; Phillips et al. 2004). Transversions accumulate more slowly than transitions in most DNA and rRNA sequences and so are less saturated, and their composition is more balanced thereby improving model fit (Brown et al. 1982; Woese et al. 1991; Phillips and Penny 2003; Phillips et al. 2004). In our example, RY recoding allowed the successful recovery of *Thermus* plus *Deinococcus*, even in the absence of *Meiothermus* (fig. 1*c*). Data recoding has also been used to ameliorate compositional heterogeneity in amino acid data and several general recoding schemes have been proposed (Hrdy et al. 2004; Kosiol et al. 2004; Susko and Roger 2007). Software is available for inferring optimal recoding schemes for a given sequence alignment (Kosiol et al. 2004; Susko and Roger 2007). The removal of fast-evolving (Brinkmann and Philippe 1999) or compositionally biased (Viklund et al. 2012; Martijn et al. 2018; Muñoz-Gómez et al. 2019) sites from sequence alignments has also been used to explore how these types of sites affected the trees recovered. For example, the removal of the 90 fastest-evolving sites from the *Deinococcus–Thermus* data set using TIGER (Cummins and McInerney 2011) reduced the level of compositional heterogeneity in the data: the χ^2^ statistic reduced from 48.9 on 1,273 sites (*P* = 2e-6) to 27.6 on 1,183 sites (*P* = 0.006). This treatment allowed the branch-homogeneous TIM2+F+I model to recover the correct *Deinococcus–Thermus* sister relationship with moderate (74%) bootstrap support.

Data recoding and site removal can have a positive effect on the accuracy of the tree recovered, but come at the cost of losing some information. The LogDet transformation (Lockhart et al. 1994; Steel 1994) and the related Paralinear Distance transformation (Lake 1994) do not assume across-tree homogeneity in nucleotide composition, and can often recover the correct tree without recoding or data editing. The LogDet is a distance measure that is based on the General Markov model of sequence evolution (Barry and Hartigan 1987) which is both nonstationary and heterogeneous; that is, in which exchangeabilities and sequence compositions can change at any time across the tree. In its original formulation, the LogDet method did not model ASRV, but removing a proportion of constant sites (i.e., the slowest evolving sites) has been shown to provide an effective partial site-rate correction (Waddell and Steel 1997; Hirt et al. 1999; Sullivan and Swofford 2001). In simulations, the LogDet is much better at recovering the correct tree under realistic conditions of compositional heterogeneity than either MP or the JC69 model (Ho and Jermiin 2004; Jermiin et al. 2004). Indeed, it was only when pronounced compositional heterogeneity was combined with very short internal branches that successful phylogenetic recovery fell below 100% in simulations using LogDet (Jermiin et al. 2004). These results are consistent with earlier observations (Lake 1994; Conant and Lewis 2001; Phillips and Penny 2003) that artifacts due to convergence in composition or unequal rate effects are exacerbated by short internal branches. As shown in figure 1*d*, rate-corrected LogDet was able to recover the clanhood of *Thermus* and *Deinococcus* in the absence of *Meiothermus ruber*.

Substitution models that allow for nucleic acid or amino acid compositions to change over the tree, and which also model ASRV, are now available within an ML or Bayesian framework (Yang and Roberts 1995; Galtier and Gouy 1998; Foster 2004; Jayaswal et al. 2005, 2011, 2014; Gowri-Shankar and Rattray 2007; Blanquart and Lartillot 2008; Groussin et al. 2013; Heaps et al. 2014; Williams et al. 2015). These models take one of three basic approaches: they implement the General Markov Model of evolution (Barry and Hartigan 1987) introduced above, in which both exchangeabilities and compositions can change at any point across the tree (Jayaswal et al. 2005, 2011); they model changes in composition at discrete breakpoints that can occur anywhere on the tree (Blanquart and Lartillot 2008); or they model composition changes at speciation events, such that different branches can have different compositions (Foster 2004; Heaps et al. 2014; Williams et al. 2015). With some exceptions such as the COaLA model (Groussin et al. 2013), in which correspondence analysis is used to identify the main axes of compositional variation, these approaches all require a substantial number of additional parameters compared with data-homogeneous models, and so to avoid problems with optimizing a large number of parameters by ML, they are now generally implemented using Bayesian Markov Chain Monte Carlo (MCMC) methods (Gowri-Shankar and Rattray 2007; Cox et al. 2008; Foster et al. 2009; Heaps et al. 2014; Williams et al. 2020). The number of compositions, their respective nucleotide or amino acid proportions, and the number and position of breakpoints on the tree are sampled by the MCMC chain.

In the node-discrete compositional heterogeneity (NDCH) model (Foster 2004) a number of independent composition vectors are arranged on the tree, generally with composition vectors shared among some branches. Composition vectors describe the long-term expected composition (frequency of A, C, G, and T states) of sequences evolving on a branch, and can be estimated from the data by ML or Bayesian methods. Standard phylogenetic models fit a single composition vector to all branches of the tree; NDCH relaxes that assumption and allows different branches to be fit by different composition vectors, so the model can accommodate (and learn about) changes in composition across the tree. The number of composition vectors is kept small to avoid overparameterization in an ML framework and for computational tractability. Figure 1*e* and *f* illustrates the performance of NDCH+G under ML on the *Deinococcus–Thermus* alignment. ML analysis of the two fixed trees under the composition-homogeneous GTR+G model does not distinguish between the two trees (AU > 0.05; Shimodaira 2002), whereas the composition-heterogeneous NDCH+G model rejects the incorrect *Deinococcus*–*Bacillus* tree. The TAMCFT (tree and model composition fit test) results indicate that the NDCH+G model, but not the GTR+G, fits the data adequately with respect to compositional heterogeneity.

In a Bayesian analysis (fig. 1*g*), model adequacy can be assessed using posterior predictive tests (Bollback 2002; Foster 2004). The χ^2^ test statistic quantifies compositional fit. To ask whether the model fits the data with respect to composition, we can compare the χ^2^ value for the real data (48.9 in this case) to a null distribution generated by simulations from posterior samples under each model. For the stationary GTR+G model all the posterior simulations had small χ^2^ values (0.43–10.2) compared with the test quantity from the original data (*P* = 0), indicating that the model does not fit the data. By contrast, simulations under the NDCH+G model generated χ^2^ values from 13.6 to 84.6, of which 35% exceeded the test quantity from the original data, meaning that the composition of this model fits the data. Inference under the NDCH2+G model, in which every branch and the root have their own composition vector (Foster 2004; Williams et al. 2020), gave similar results (fig. 1*g*). Thus, use of a branch-heterogeneous model (NDCH+G or NDCH2+G) improved model fit (as assessed by marginal likelihoods estimated using the Stepping Stone method; Xie et al. 2011) and model adequacy (as assessed by posterior predictive tests), and allowed inference of the correct *Deinococcus+Thermus* tree.

### Compositional Heterogeneity across Sites Is Also a Common Feature of Molecular Data

Many of the variable positions in proteins can only tolerate a limited number of different amino acids, because of structural and functional constraints (Miyamoto and Fitch 1995). As a consequence, most site changes are within classes (acidic, aromatic, basic, polar, or nonpolar amino acids: Dayhoff et al. 1978), rather than between them. Models that recognize these site-specific biochemical preferences generally fit data much better (Koshi and Goldstein 1998; Lartillot and Philippe 2004; Lartillot et al. 2007; Quang et al. 2008; Wang et al. 2008; Lartillot 2015; Williams et al. 2020) than site-homogeneous empirical amino acid replacement models like LG (Le and Gascuel 2008) and WAG (Whelan and Goldman 2001). A full Bayesian treatment of site compositional variation is provided by the CAT model (Lartillot and Philippe 2004; Lartillot et al. 2007, 2013) which is often the best-fitting of the available substitution models for proteins. The CAT model effectively clusters the sites of the alignment into biochemically specific categories, each of which is described by its own amino-acid profile of equilibrium frequencies. The number of different compositions, their constituent proportions, and the assignment of alignment sites to compositions are sampled during a Bayesian MCMC analysis. In principle, each site in the alignment might merit its own compositional profile under CAT. However, a Dirichlet process prior is used to tune the required number of distinct compositional profiles to match the level of site compositional heterogeneity observed in the data. One potential drawback is that Bayesian analyses using CAT may sometimes take a long time to converge for large data sets, and a lack of convergence has also been reported for some alignments (Da Cunha et al. 2017; Whelan et al. 2017).

The CAT model is reported to be relatively resistant to LBA compared with site-homogeneous models (Lartillot and Philippe 2004; Lartillot et al. 2007; Williams et al. 2020). However, the CAT+GTR+G model was unable to recover Microsporidia plus Fungi (M+F) when used to analyze concatenated SSU and LSU rRNA sequences in the presence of archaeal and bacterial outgroup sequences (Cox et al. 2008; Foster et al. 2009). Applying the same model to a concatenation of 45 conserved proteins involved in DNA replication, transcription, or translation, from all three domains of life, also failed to recover M+F; the Microsporidia were recovered at the base of eukaryotes (“Microsporidia early”) with strong posterior probability values (≥95% support). However, after removing the prokaryotic outgroups M+F was recovered (Cox et al. 2008). As mentioned earlier, the conserved proteins used in these studies show marked across-tree compositional heterogeneity (Cox et al. 2008), suggesting that a failure to model or mitigate this heterogeneity may be part of the problem for CAT+GTR+G in the presence of the outgroups (Cox et al. 2008). To investigate further, the full amino acid data set was recoded according to the six “Dayhoff groups” of chemically related amino acids that commonly replace one another (Hrdy et al. 2004; Susko and Roger 2007). Analysis of these recoded data using the CAT+GTR+G model recovered M+F even when the prokaryotic outgroups were included in the tree (Cox et al. 2008). The recovery of M+F using CAT+GTR+G on Dayhoff-recoded data and including an increased taxonomic sampling of outgroup Archaea, was also reported by Foster et al. (2009).

The CAT model accounts for variation in composition across sites but not across branches, in that the same site-specific composition is applied to all of the sites in the same column of the alignment. A model that could accommodate both features of the data at the same time would be extremely useful. An extension of CAT that allows for changing compositions across the tree, termed CAT-BP (breakpoint), has been published (Blanquart and Lartillot 2008). This allows the joint modeling of site- (CAT) and branch- (BP) heterogeneity and hence it can potentially recover the correct tree in situations where individual modeling of one of these two properties of data does not. For example, CAT-BP correctly recovered the monophyly of insects from a concatenation of mitochondrial proteins for which CAT, BP, and homogeneous GTR each incorrectly placed the fast-evolving, AT-rich honeybee sequences within the Chelicerates, a distant clade of arthropods (Blanquart and Lartillot 2008). Unfortunately, the increased sophistication of CAT-BP apparently comes at the cost of tractability, since MCMC convergence is an even greater challenge than with CAT. The development of a scalable and efficient method for joint modeling of branch- and site-heterogeneity remains a major challenge for phylogenetics, and a reminder of the potential limitations of even the best methods currently available.

As a computationally efficient and more scalable alternative to the Bayesian CAT model, several authors have investigated models that have a fixed number of site compositions precomputed from existing sequence alignments (Quang et al. 2008; Wang, Li, et al. 2008; Schrempf et al. 2020). These fixed compositions are taken to represent general patterns in sequence data, similar to the logic underpinning the fixed exchange rates between amino acids in empirical single matrix models such as LG (Le and Gascuel 2008). Tree inference then involves estimating far fewer parameters than the full CAT model because the number of composition vectors and their constituent amino acid proportions are fixed; the alignment is treated as evolving under a mixture model for which only the weights of each composition must be inferred. This makes analysis tractable under maximum likelihood, and efficient implementations of these models that scale to reasonable numbers (100 s) of taxa and alignment lengths (1,000–10,000s of sites) have been implemented in IQ-TREE 2 (Minh et al. 2020) and RAxML (Kozlov et al. 2019).

The first models of this type were the class frequency (cF) mixture model (Wang, Li, et al. 2008), which included four fixed site compositions and one general composition estimated from the alignment of interest, and the C10–C60 (CXX) models, with 10–60 fixed site compositions (Quang et al. 2008). For very large data sets, computational efficiency can be increased further by estimating a fixed, site-specific composition for each site in the alignment, thereby avoiding the need to compute the likelihood at each site for each mixture component (Wang et al. 2018). Recent developments include an ML method (MAMMaL) for estimating site compositions directly from the alignment of interest (Susko et al. 2018) and the universal distribution mixture (UDM) models (Schrempf et al. 2020), which comprise precomputed models with up to 512 site compositions inferred using a distinct clustering approach (EDCluster) for estimating site profiles from large sets of sequence alignments. Like the CXX models, UDM models use flat (Poisson) exchange rates among amino acids, because the single gene alignments used for training the models are individually too short to infer both compositions and exchangeabilities. This is a compromise because rates of change among pairs of amino acids vary in real data, although some of that signal is captured in the site profile compositions (Schrempf et al. 2020).

Both MAMMaL and the UDM models show improved model fit and performance compared with the CXX models. For example, both approaches (MAMMaL with 20–30 site profiles; Susko et al. 2018 and UDM+G with 128 and 256 components; Schrempf et al. 2020) recovered the correct Microsporidia+Fungi tree from the previously discussed 133-gene data set (Brinkmann et al. 2005; Wang et al. 2009), as does the full CAT+GTR model, under conditions in which the C60+G, the LG+G, and the WAG+G models recovered the incorrect “Microsporidia early” tree (Schrempf et al. 2020).

### Model Fit and Model Adequacy

There are currently two approaches to choosing a model for analyzing molecular sequence data. One looks at the relative fit of a suite of models to a data set and chooses the best fitting model of those tested (Posada and Buckley 2004; Darriba et al. 2012). The other approach tests for model adequacy by investigating whether the data are likely to have arisen under the model (Goldman 1993; Bollback 2002; Foster 2004); reviewed in Shepherd and Klaere (2019). In the first approach, the comparison of AIC or BIC scores can be used to select the best model for a data set from a set of candidates, but the winning model may still not fit the data very well: it is simply the best of the models that were evaluated. This procedure is implemented in commonly used packages such as jModelTest, (Darriba et al. 2012), ModelFinder (Kalyaanamoorthy et al. 2017), and ModelTest-NG (Darriba et al. 2020).

Bayesian posterior predictive simulations (Bollback 2002) provide a useful way of testing model adequacy, and whether data simulated under a model are similar to some property of the empirical data. These analyses involve computing a statistic of interest, such as a χ^2^ statistic for compositional homogeneity (Foster 2004) or the mean number of different character states per site (Lartillot et al. 2009), on the observed data and on a large number of equally sized data sets simulated under the model, using the parameter configurations and tree sampled at each step in an MCMC analysis. The simulated data are used to calculate a null distribution for the test statistic that averages over the uncertainty in the parameters. The observed value for the real data can then be compared with this null distribution to determine if the observed data could have plausibly been generated under the model. For example, posterior predictive simulations were used to demonstrate that the GTR model commonly used to analyze rRNA data, does not produce sequences with the diversity of nucleotide composition observed in the *Deinococcus*/*Thermus* SSU rRNA data set (Foster 2004 and fig. 1*g*), or in SSU and LSU rRNA sequences sampled from across the tree of life (Cox et al. 2008). In both of these cases the use of the NCDH composition-heterogeneous model, which fits the data better for composition, supports a different tree to the one recovered by the GTR model. For the *Thermus*/*Deinococcus* data set, the GTR model recovered the incorrect “attract tree” whereas the NDCH model supports the sisterhood of *Thermus* and *Deinococcus* (fig. 1). In the analysis of SSU and LSU rRNA sequences from across the tree of life, the poorly fitting GTR model produced a classic 3-domains tree wherein eukaryotes are a separate group (Woese et al. 1990), whereas the NDCH analyses placed eukaryotes within the Archaea (Cox et al. 2008; Foster et al. 2009). Identifying which of these two trees is correct is important for understanding how eukaryotes evolved from prokaryotes and for identifying what kind of cell might have hosted the mitochondrial endosymbiont.

### Where Do Eukaryotes Fit in the Tree of Life?

It is currently thought that the last common ancestor of eukaryotes already possessed mitochondria (Martin et al. 2015; Roger et al. 2017). This suggests that endosymbiosis between a bacterial endosymbiont and a host cell was a key step in eukaryogenesis. There has been a long-standing debate about the nature and phylogenetic position of the host cell (reviewed in Doolittle [2020]). Trees based upon analyses of the relatively small number of genes that are conserved in all three major groups of life have been central to this debate. Early analyses of ribosomal RNA and other broadly conserved genes using simple models recovered an unrooted tree (e.g., figure 4 in Woese [1987]) in which bacteria, archaea, and eukaryotes were recovered as three separate groups. The unrooted tree did not resolve the order of divergence of the three groups and so allowed for the possibility that they might be of equal antiquity (Woese and Fox 1977; Kurland et al. 2006). The universal SSU rRNA tree was subsequently rooted using external data from analyses of ancient elongation factor and ATPase paralogs that suggested the root was on the bacterial branch (Gogarten et al. 1989; Iwabe et al. 1989; Woese et al. 1990). In the rooted tree (Woese et al. 1990), the eukaryotes and Archaea are sister taxa with a common ancestor that is not shared with bacteria, but which parsimony would suggest was already a prokaryote. The three major groups were subsequently renamed “domains” and the rooted three domains (3D) tree was adopted in textbooks and reviews as the main framework for understanding the diversity of cellular life.

Despite its prominence, it was soon suggested (Lake 1988) that the 3D tree was an artifact of long-branch attraction (LBA) between bacteria and eukaryotes, the two longest branches in the tree. In a series of papers (Lake et al. 1984; Lake 1988, 1994; Rivera and Lake 1992), Lake developed the hypothesis that eukaryotes were the sister lineage to a specific group of Archaea that he called Eocytes (Lake et al. 1984); the same group later named Crenarchaeota by Woese et al. (1990). Lake’s hypothesis subsequently received support from analyses of ancient duplicated genes (Baldauf et al. 1996; Hashimoto and Hasegawa 1996) and from analyses of rRNA and RNA polymerases using models accounting for ASRV (Tourasse and Gouy 1999), but it was still overshadowed by the canonical 3D tree (reviewed in Williams et al. [2013]; McInerney et al. [2014]). However, recent phylogenomic analyses of conserved genes using methods like CAT have recovered trees (Raymann et al. 2015; Spang et al. 2015; Zaremba-Niedzwiedzka et al. 2017; Williams et al. 2020) that support a version of Lake’s eocyte hypothesis, when allowance is made for the expanded sampling of Archaea now available (Doolittle 2020). To distinguish it from the 3D tree, this tree is now generally referred to as the two-domains (2D) tree, because the basal split identifies Bacteria and Archaea as the two candidate primary domains of life (sensu Woese et al. 1990).

Despite the growing support for the 2D tree discussed above, some recent analyses of conserved genes using the homogenous LG model have recovered the 3D tree (Da Cunha et al. 2017, 2018), and it is interesting to explore why. It has been suggested (Tourasse and Gouy 1999) that the 2D tree is difficult to recover using simple models, because it requires placing a long branch (the eukaryotic stem) within a short-branching clade (the Archaea). By contrast, in the 3D tree, the two longest branches (the bacterial and eukaryotic stems) are joined together, potentially due to LBA. Previous work (Lartillot et al. 2007; Williams et al. 2020) has shown that, when the data is site-heterogeneous, as most molecular data are, overly simple models can be vulnerable to LBA because they can underestimate the number of convergent changes. When taxa share the same amino acid at an alignment site, two explanations are possible: either they inherited that state from a common ancestor, or they arrived at the same amino acid by convergent evolution from different starting points. Site-specific information is critically important for distinguishing these two possibilities. At a site where all 20 amino acids are observed, the first explanation seems more likely, but convergence is increasingly favored as the number of possible amino acids at the site decreases. Simple site-homogeneous substitution models, in which the evolutionary process is averaged over the alignment, are potentially susceptible to LBA artifacts because they ignore this site-specific context. Consistent with this hypothesis, the use of the CAT model to reanalyze the data recently used to support the 3D tree using LG (Da Cunha et al. 2017, 2018) instead recovered a 2D tree with strong support (Williams et al. 2020).

**Fig. 2.**
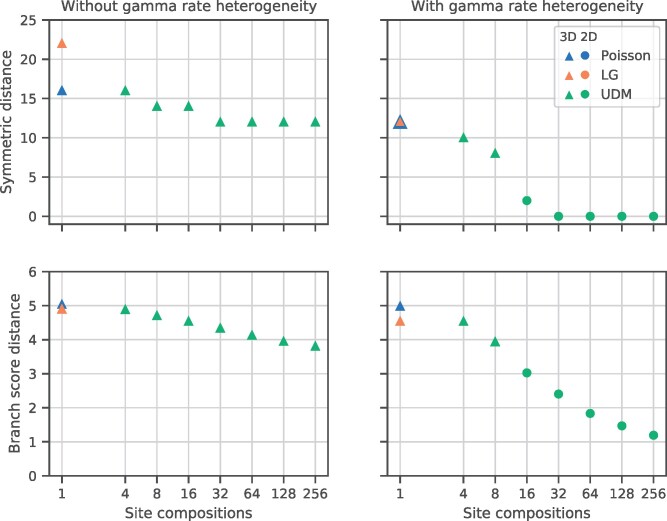
—Simulations to evaluate the difficulty of recovering the 2D and 3D trees using simple and more complex phylogenetic models. We simulated site-heterogeneous amino acid sequence alignments under site compositions obtained from the HOGENOM database (Dufayard et al. 2005) on 2D tree and 3D trees, with the alignment dimensions and simulation trees taken from a recent empirical study (Williams et al. 2020). We then evaluated the ability of a set of increasingly complex substitution models to recover the true simulation tree. “Poisson” denotes a model with uniform exchangeabilities between amino acids and a single composition vector; “LG” denotes a model with LG exchangeabilities (Le and Gascuel 2008) and a single composition vector; UDM denotes a series of Universal Distribution Mixture models with uniform exchangeabilities and four or more site compositions. When data were simulated on a 3D tree, all analyses (both simple and complex models, with and without ASRV) recovered the correct 3D tree (not shown). When data were simulated on the 2D tree, the results depended on the model used to analyze the data. If ASRV was not modeled (no gamma distribution, left panels), then all analyses recovered the incorrect 3D tree. When ASRV was modeled, models with one to eight site compositions (Poisson, LG, UDM model with four or eight components) recovered an incorrect 3D tree, whereas models with 16 or more site compositions recovered the correct 2D tree. Whether the data were simulated on a 2D or a 3D tree, branch lengths and within-domain relationships were more accurately estimated under the more complex models, as indicated by the reduced symmetric and branch length distances to the simulation tree with an increasing number of site compositions. The results indicate that the 2D is intrinsically more difficult to recover than the 3D tree, and that modeling of both site rate and site compositional heterogeneity may be necessary to recover the true tree for difficult phylogenetic problems.

To explore further, we used simulations to investigate the role of site heterogeneity in recovering the 2D and 3D trees in a controlled setting where the true (simulation) tree is known. To do this, we simulated data with among-site rate (ASRV) and/or composition heterogeneity as two common potential causes of LBA (see Materials and Methods). We then evaluated the ability of simple and more complex models to recover the true tree that generated the data (fig. 2). In these simulations, we used empirical 2D and 3D trees, including branch lengths, from a recent study (Williams et al. 2020), and simulated data sets similar in size (7,000 sites) to the real alignment used in those analyses. To capture realistic levels of site compositional heterogeneity, we simulated data under site compositions obtained from the HOGENOM database (Dufayard et al. 2005) on both 2D and 3D trees. The simulated data are likely to recapitulate the compositional variation of real sequence data, because the patterns of site heterogeneity are based on a sample of over 1 million gene family alignments. We then analyzed the simulated data with a set of UDM models with constant (Poisson) exchangeabilities combined with 1, 4, 8, 16, 32, 64, 128, or 256 site compositions, with and without ASRV. We also analyzed the simulated data with the LG model (Le and Gascuel 2008) which assumes a single site composition, with and without ASRV. As mentioned above, the LG model has featured heavily in recent discussions of which tree, 3D or 2D, is best supported by molecular data (Da Cunha et al. 2017, 2018; Spang et al. 2018; Williams et al. 2020).

When site-heterogeneous data were simulated on a 2D tree, inference under the LG model always recovered an incorrect 3D tree, consistent with it being susceptible to LBA when faced with this common feature of real data. By contrast, all inferences under the more complex UDM models with 16 or more site compositions (Schrempf et al. 2020) recovered a 2D tree (fig. 2). The simulations also confirm previous work that modeling ASRV is particularly important for obtaining the correct tree when long branches are present. Thus, all analyses of the 2D-simulated data without ASRV recovered a 3D tree, regardless of the model used. These results demonstrate that joint modeling of both site rate and site compositional heterogeneity is necessary to recover the true 2D tree for these data. By contrast, when data were simulated on a 3D tree, all of the models, with or without ASRV, recovered the 3D tree. Interestingly, and regardless of whether the data were simulated on a 2D or a 3D tree, the branch lengths and within-domain relationships were more accurately estimated under the more complex models, as indicated by the reduced symmetric and branch length distances to the simulation tree with an increasing number of site compositions (fig. 2).

**Fig. 3.**
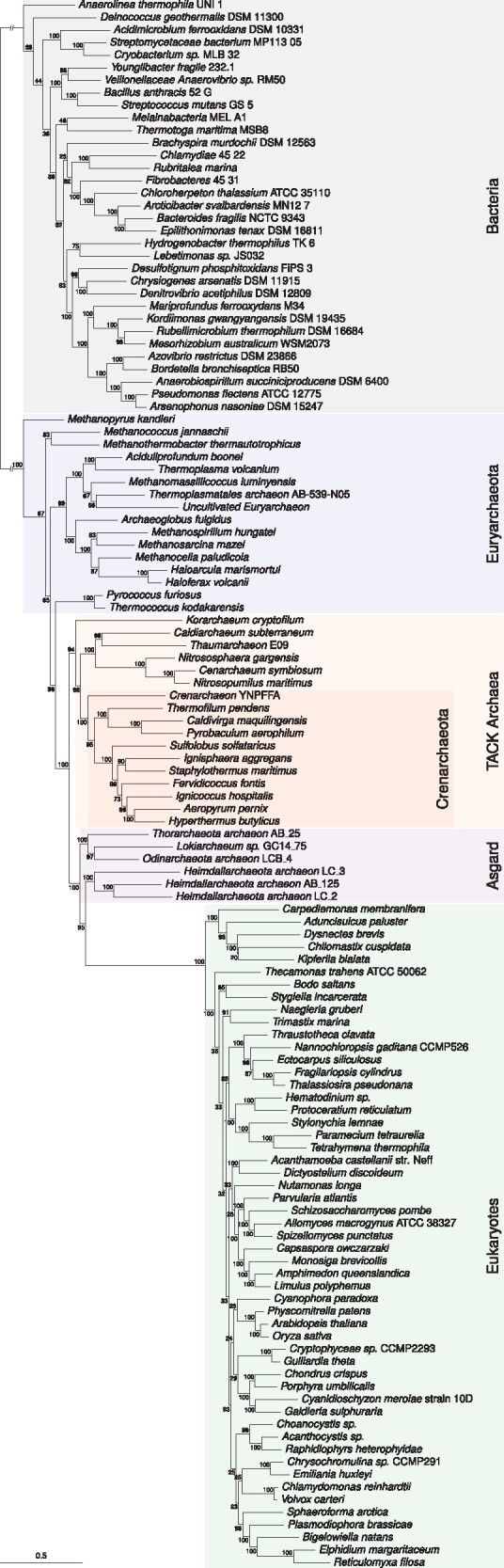
—Analysis with a universal distribution model (UDM 128+G) to investigate the placement of eukaryotes within Archaea. ML phylogeny inferred from a concatenation of 27 broadly conserved marker genes under the UDM 128+G model. The analysis places the eukaryotic nuclear lineage within Asgard archaea as the sister lineage to Heimdallarchaeota with high (95%) ultrafast bootstrap support. Branch lengths are the expected number of substitutions per site. To improve the legibility of the internal structure of the trees, the branch separating bacteria and archaea is shown at 1/10th its true size.

Given the results of our simulations, we reanalyzed an empirical data set used to investigate the relationships between bacteria, archaea, and eukaryotes with the UDM 128+G model (Schrempf et al. 2020). The data set comprises a concatenation of 27 universal genes (6,419 amino acid sites) for 125 taxa including bacteria, archaea, and eukaryotes. These 27 genes were chosen because they were present in two out of three marker gene data sets used in recent analyses of the tree of life (Spang et al. 2015; Da Cunha et al. 2017; Williams et al. 2020). Consistent with recent work suggesting that the 2D tree is the best-supported tree for these data (Williams et al. 2020), we recovered a maximally supported (100% ultrafast bootstrap) 2D tree (fig. 3). In this tree, the eukaryotes were the sister group to the Heimdallarchaeota, a lineage within the recently discovered Asgard archaea that form a clade with eocytes/Crenarchaeota in the archaeal tree (fig. 3).

## Conclusions

In this review, we have focused on the analysis of molecular sequence data to make phylogenetic trees for ancient relationships using substitution models. Our aim has been to use three classic case studies, which we and many other labs have worked on, to demonstrate that the choice of model, and how well it fits the often very complicated data being analyzed, can have a profound effect on which tree is recovered. Hence, some apparently well-supported and influential trees which were made with commonly used but overly simple models are now known to have been incorrect and to have misled thinking about evolutionary relationships. In the context of making phylogenies, standard measures of statistical support such as bootstraps and posterior probabilities only measure uncertainty in estimates assuming a specific evolutionary model, and hence may be an unreliable guide to the accuracy of the inferred tree. Methods that directly evaluate the adequacy of models and trees (Goldman 1993; Bollback 2002; Jermiin et al. 2020) have been developed but are not yet widely used.

We have not discussed genome-scale evolutionary processes such as incomplete lineage sorting, gene duplications, losses, and horizontal transfers, all of which are common phenomena that potentially affect the quality of data sets and tree topologies. Methods are being developed to account for disagreements among the large numbers of gene trees that can be produced from genome-scale data, and to harness that discord to learn about evolutionary history. These include methods based on the multispecies coalescent (Bouckaert et al. 2014; Höhna et al. 2016; Zhang et al. 2017, 2020) or explicit models of gene duplication, transfer, and loss (Szöllõsi et al. 2013; Jacox et al. 2016; Bansal et al. 2018; Morel et al. 2020). These methods can potentially bring much more data to bear on interesting problems including phylogenomic rooting, the quantification of vertical and horizontal gene flows, ancestral genome reconstructions, and the inference of endosymbioses from genome data. However, to the extent that these methods use source trees made with overly simple models of the substitution process, the issues we discuss that can affect the accuracy of individual trees are directly relevant. The CXX and UDM models may be useful in this context because they potentially allow large numbers of single-gene trees to be inferred under tractable, computationally efficient models that account for site heterogeneity without a very large number of additional parameters.

George Box is famous for suggesting that models that seek to represent the real world are always wrong, yet they can still be illuminating and useful. Although new models have been developed that fit some features of real data much better than previous models, the patterns in data can be complicated and can vary among sites, genes, and lineages. As a result, there can be complex interactions between different confounding factors in data, with an outcome that is difficult to diagnose or predict a priori. None of the currently available models can reasonably be expected to fit such data perfectly, and posterior predictive simulations for individual models often show that the fit between model and real data is inadequate. In this situation, the question is whether or not the model is sufficient to recover useful information about the particular relationships that are of interest to the study. As our examples attest, carefully chosen models can often recover what are believed to be the correct relationships in the face of ASRV, variable lineage-specific rates, site-specific composition effects, and changing across-tree nucleotide or amino acid composition. Conversely, there is overwhelming evidence that the use of overly simple models to analyze real data will often fail to recover the correct tree.

Given that no current method deals with all of the different types of heterogeneity in real data equally well, exploring the stability of trees using methods that focus on different properties of data can be helpful for identifying where problems might lie. For example, making trees based on amino acid or nucleotide composition is a simple way to identify sequences which have the potential to group together because of their shared composition rather than their shared history. Since some models are demonstrably better at dealing with this type of convergence than others, using them should probably be more routine than it currently is. In our review, we have used the *Deinococcus*–*Thermus* example to illustrate how trees can radically change when dramatic compositional heterogeneity is ignored, mitigated, or modeled. Across-tree compositional heterogeneity appears to be a pervasive feature of most molecular data, and so is potentially a common source of model misspecification that is still often ignored in the published literature.

Even the best available models can fail to recover accurate ingroup relationships, when long-branch outgroups are present and internal branches are short. The difficulties in recovering the relationship between Microsporidia and Fungi demonstrate just how challenging this can be, and the literature is littered with examples of other difficult “long-branch problems” (Philippe et al. 2011; Gouy et al. 2015). In these cases, agreement between models for a particular tree topology may simply reflect their shared inadequacy at modeling the complexities of the data. As we discuss in the main text, methods that explicitly accommodate site-specific compositional heterogeneity appear to be better than others at dealing with long branches. But as was shown for the position of honeybees within insects (Blanquart and Lartillot 2008), they can still sometimes fail to fully mitigate the problem of LBA, because they do not model across-tree compositional heterogeneity. In cases where LBA to long-branch outgroups is suspected, repeating the analyses in the absence of outgroups can often provide an informative check on the stability of ingroup relationships.

Long branches and data heterogeneity are particularly prominent features of universal trees investigating the relationships between major groups and the domains of cellular life, so it is unsurprising that the topologies of these trees have greatly changed as models have improved. The Archezoa hypothesis (Cavalier-Smith 1987) proposed that some eukaryotes might primitively lack mitochondria and was founded on early rRNA and protein trees and the apparent absence of cytological and biochemical evidence for mitochondria in early-branching protist lineages like Microsporidia. But more data and better models have rearranged eukaryotic relationships to the extent where the latest eukaryotic tree (Burki et al. 2020) bears little resemblance to the classical rRNA tree. In the new tree, Microsporidia group with Fungi and there is no compelling evidence to argue that any of the other former archezoans branch at the base of eukaryotes, especially since the root of the eukaryotic tree is still uncertain (Burki et al. 2020). Since all of the best-studied former archezoans are also now known to contain highly reduced versions of mitochondria (Embley and Martin 2006; Martin et al. 2015; Roger et al. 2017), the idea that some eukaryotes primitively lack mitochondria has fallen out of favor for lack of good candidates. As a consequence, there seems to be no compelling reason to assume that eukaryotes, as we now define them, must have predated the mitochondrial endosymbiosis.

The impact of improved methods of analysis and more data on current ideas about the topology of the tree of life, and the place of eukaryotes within that tree, has been dramatic. Here, the potential for substitutional saturation and overwriting over vast time scales potentially amplifies the difficulties of obtaining a robust hypothesis of relationships from already complex data. At present, the best available methods support a two-domains tree wherein eukaryotes originate from within the Archaea, consistent with some formulations of Lake’s eocyte hypothesis (Rivera and Lake 1992; Williams et al. 2013, 2020; Doolittle 2020). Universal trees generally assume a root on the bacterial branch, but this rooting owes much of its prominence to the early days of phylogenetic analysis using simple models (Gogarten et al. 1989; Iwabe et al. 1989; Woese et al. 1990; Brown and Doolittle 1995; Zhaxybayeva et al. 2005; Gouy et al. 2015). The issues we discuss about long branches and the complexity of data suggest that we should be cautious in claiming that we have a robust estimate for the root of the universal tree, especially given its fundamental importance for understanding the earliest period of cell evolution.

Given the complexities of real data and the limitations of even the best models, it is no surprise that phylogenetic inferences of ancient relationships have been so tentative and challenging. However, progress has been made and there exists a robust statistical toolbox that can be used to evaluate models and support for phylogenetic hypotheses from molecular data. Congruence or consilience (Whewell 1840; Darwin 1859) between different types of data should also be used to test or support inferences. For example, a close relationship between *Thermus* and *Deinococcus* is supported by the complex lipid and cell wall characters that they share. The relationship between Microsporidia and Fungi is supported by the discovery of a diversity of environmental lineages that branch between canonical fungi and microsporidia and display a mixture of ancestral and derived characters (James et al. 2013; Bass et al. 2018). Independent data that speak decisively to the relationship between eukaryotes and archaea are more elusive, but the discovery of the new Asgard archaea, which contain more of the genes for proteins formerly claimed to be eukaryote-specific (Spang et al. 2015; Zaremba-Niedzwiedzka et al. 2017; Imachi et al. 2020) is consistent with an archaeal origin for at least some of the building blocks of eukaryotic complexity.

## Materials and Methods

### 2D/3D Simulations and Analysis with UDM Models

Two trees T3D and T2D exhibiting a 3D and 2D topology were obtained from Da Cunha et al. (2017), and Williams et al. (2020), respectively. Both trees had been inferred from the same 35 gene matrix (Da Cunha et al. 2017), albeit with different models. T3D was inferred by the LG model (Le and Gascuel 2008) with discrete gamma rate heterogeneity (Yang 1994) in PhyML (Guindon et al. 2010), and T2D by the CAT model (Lartillot and Philippe 2004) with GTR exchangeabilities (Tavaré 1986) and four-category discrete gamma rate heterogeneity in Phylobayes-MPI (Lartillot et al. 2013).

Alignments with 7,000 amino acid columns were simulated along the trees using a model with varying compositions of amino acids across sites. To this end, for each site, a random composition of amino acids was sampled from a set of compositions previously obtained from an analysis (Schrempf et al. 2020) of the HOGENOM database (Dufayard et al. 2005). Uniform exchangeabilities (Poisson model; Felsenstein 1981) were used throughout. For each tree, an alignment with discrete gamma rate heterogeneity (four categories and a gamma distribution parameter of 0.935) and without rate heterogeneity was simulated. The ELynx software package (https://github.com/dschrempf/elynx, last accessed April 5, 2021) was used for these simulations.

Trees were inferred from the four simulated alignments with IQ-TREE 2 (Minh et al. 2020). The model of rate heterogeneity (four-category gamma) used for simulating the alignment was also used for inference. We used the Poisson, LG (Le and Gascuel 2008) and UDM (Schrempf et al. 2020) models for inference. For all inferences, the ultrafast bootstrap (Hoang et al. 2018) with 1,000 samples was used. Finally, each reconstructed tree was analyzed for compatibility with the 2D or the 3D topology. 
